# Re-emergence of *Mycoplasma pneumoniae* before and after COVID-19 pandemic in Germany

**DOI:** 10.1186/s12879-025-10657-4

**Published:** 2025-03-06

**Authors:** Frederike Waldeck, Tobias Siegfried Kramer, Sebastien Boutin, Jens Matten, Jan Kramer, Jan Rupp

**Affiliations:** 1 Infectious Diseases Clinic, University Hospital Schleswig-Holstein, Campus Luebeck, Ratzeburger Allee 160, 23538 Luebeck, Germany; 2 LADR Laborverbund Dr. Kramer & Kollegen, Geesthacht, Germany; 3 Institute for Hygiene and Environmental medicine Charité-Universitätsmedizin Berlin, Berlin, Germany; 4 Institute of Medical Microbiology, University hospital Schleswig-Holstein, Campus Luebeck, Luebeck, Germany; 5 LADR GmbH MVZ Nordwest, Schuettorf, Germany

**Keywords:** *Mycoplasma pneumoniae*, Pneumonia, Epidemiology

## Abstract

**Background:**

*Mycoplasma pneumoniae* (*M. pneumoniae*) is a common pathogen of community-acquired pneumonia (CAP). Epidemics occur every 3–7 years especially in pediatric patients. We collected data from a large laboratory network in Germany to define the epidemiological dynamics in the pre- and post-COVID-19 pandemic period.

**Methods:**

In this retrospective cohort study we included all patients that obtained targeted or multiplex PCR for *M. pneumoniae* from nasopharyngeal swabs, sputum or bronchoalveolar fluids from 2015 to 2024. Demographic data (age, sex, place of residence, in- or outpatient status) were compared between *M. pneumoniae* positive and negative patients and co-infections with bacterial or viral pathogens analyzed.

**Results:**

We screened 38,204 patients for *M. pneumoniae*. We identified 1448 cases (3.8%) of *M. pneumoniae* (48.8% females). Pediatric patients ≤ 18 years represented 75.7% of *M. pneumoniae* patients and 2.3% were ≥ 60 years. Incidence of *M. pneumoniae* increased in fourth quartile 2015 (16.2%), second quartile 2018 (14.8%) and fourth quartile 2023 (13.4%). No cases were detected during COVID-19 pandemic 2021. Young age (aOR 0.98 95%-CI 0.97–0.98), outpatient status (aOR 0.56 95%-CI 0.43–0.71) and year of testing (OR dependent on year of testing) were predictors of *M. pneumoniae* detection in multivariate analysis (*p* < 0.001). We observed a significant increase in outpatients with *M. pneumoniae* after COVID-19 pandemic (86.7 vs. 96.5%, p = < 0.001, aOR 0.25, 95% CI 0.15–0.4).

**Conclusions:**

Empirical treatment of CAP patients often does not include coverage of *M. pneumoniae*. A more thorough implementation of available surveillance data into clinical routine, respective therapies could be adapted more quickly during epidemic outbreaks of *M. pneumoniae* infections.

**Supplementary Information:**

The online version contains supplementary material available at 10.1186/s12879-025-10657-4.

## Background

*Mycoplasma pneumoniae* (*M. pneumoniae*) is one of the most common bacterial pathogens of community-acquired pneumonia (CAP). Studies from North America and Europe estimate that *M. pneumoniae* are accountable for 10–40% of CAP in children and 4–8% in adults during endemic periods [1, 2]. *M. pneumoniae* is most frequently detected during winter months. Incidences can rise to 40% in adults during epidemics which occur every 3–7 years [3]. *M. pneumoniae* can cause asymptomatic infection, mild, self-limiting disease or severe CAP [1, 4]. Co-infections with respiratory viruses are common in children [1]. Approximately 25% of patients have extrapulmonary manifestations including pericarditis, endocarditis, hepatitis, arthritis, encephalitis, aseptic meningitis, otitis and thrombosis [5]. Furthermore, *M. pneumoniae* is commonly linked to mucocutaneous disease including erythema multiforme, erythema nodosum, Stevens-Johnson syndrome and mucositis. Infections with *M. pneumoniae* increase the risk of childhood asthma and exacerbation of chronic lung disease and are a common cause of severe CAP in adults [4].

*M. pneumoniae* cannot be detected in routine microbiological diagnostics but special media for bacterial growth or testing by polymerase chain reaction (PCR) are needed. Furthermore, serology can identify *M. pneumoniae* infection if a 4-fold increase in titer or seroconversion is detected. Therefore *M. pneumoniae* is presumably underdiagnosed. No reporting obligation exists for *M. pneumoniae* in Germany. During COVID-19 pandemic few cases of *M. pneumoniae* were reported from 2020 to 2022. Few data have been reported so far on rates of *M. pneumoniae* after the pandemic: Recent data show an increase of *M. pneumoniae* cases since 2023 in the Europe and China [6–10]. We aimed to characterize *M. pneumoniae* detection before and after COVID-19 pandemic in Germany.

## Methods

We conducted a retrospective cohort study including all patients which were identified in the laboratory data base to be tested for *M. pneumoniae* from respiratory samples from January 2015– May 2024. Samples were sent to and collected at two large microbiology laboratories of the LADR Laboratory group Dr. Kramer & Colleagues that perform diagnostics for > 20,000 physicians and > 400 inpatient clinics especially from the North Western region of Germany. There were no exclusion criteria. If more than one sample per patient was sent to the laboratory within 72 h, one sample was randomly selected and included in the analysis. Respiratory material included nasopharyngeal swabs, sputum and bronchoalveolar lavage fluids. Baseline characteristics of patients were date of sampling, age, sex, postal code and data on outpatient or inpatient sampling. Detected co-infection with other respiratory viruses and bacteria on multiplex PCR were analyzed. The study was approved by the ethics committee of the university of Luebeck according to the declaration of Helsinki (number 2024-897167). Anonymous data was used, no consent to participate was required. COVID-19 pandemic was dated to 11.03.2020–5.5.2023 according to the world health organization`s definition as a pandemic.

### Microbiology

All analyses were performed at LADR. Three different PCRs were used to detect *M. pneumoniae* according to manufacturer’s specification (supplementary Table 1): two multiplex PCRs (Allplex™ PneumoBacter Assay/Allplex™ RV Essential Assay by Seegene; Panel 1 and NxTAG^®^ Respiratory Pathogen Panel Test by Luminex; Panel 2) including several bacterial and viral pathogens and a *M. pneumoniae* targeted PCR (ModularDx Kit M. pneumoniae, TibMolBiol). PCRs were performed according to the respective technical instructions in the different LADR laboratories upon request of the sending physician. Both multiplex PCR panels included *Mycoplasma pneumoniae*, adenovirus, influenza virus A/B, human metapneumovirus, parainfluenza virus 1–4, respiratory syncytial virus, *Chlamydophila pneumoniae* and *Legionella pneumophila.* Panel 1 further included human rhinovirus A/B/C, *Bordetella pertussis / parapertussis*,* Haemophilus influenzae and Streptococcus pneumoniae.* Panel 2 also included human bocavirus, coronavirus (229E, HKU1, NL63, OC43) and rhino-/enterovirus. SARS-Cov-2 was included in panel 2 since 2022.

### Statistics

Statistical analysis was performed using Jamovi (version 2.3.28.0) and R (version 4.2.2.). Statistical tests were performed without imputation of missing values. *P*-value < 0.05 was considered statistically significant. Normal distribution was analyzed using Shapiro-Wilk test and q-q analysis. *P*-value was calculated using Mann-Whitney U-test and Wilcoxon test for nonparametric data where appropriate. Logistic regression using a generalized linear binomial model including age, gender, in/out-patient status and the year of testing as co-variables was used to obtain odds ratio associated to *M. pneumoniae* positivity using the package epiDisplay. Collinearity was tested using Variance Inflation Factor (VIF) and all included variables were independent (VIF range: 1.00-1.14).

## Results

**Fig. 1:**
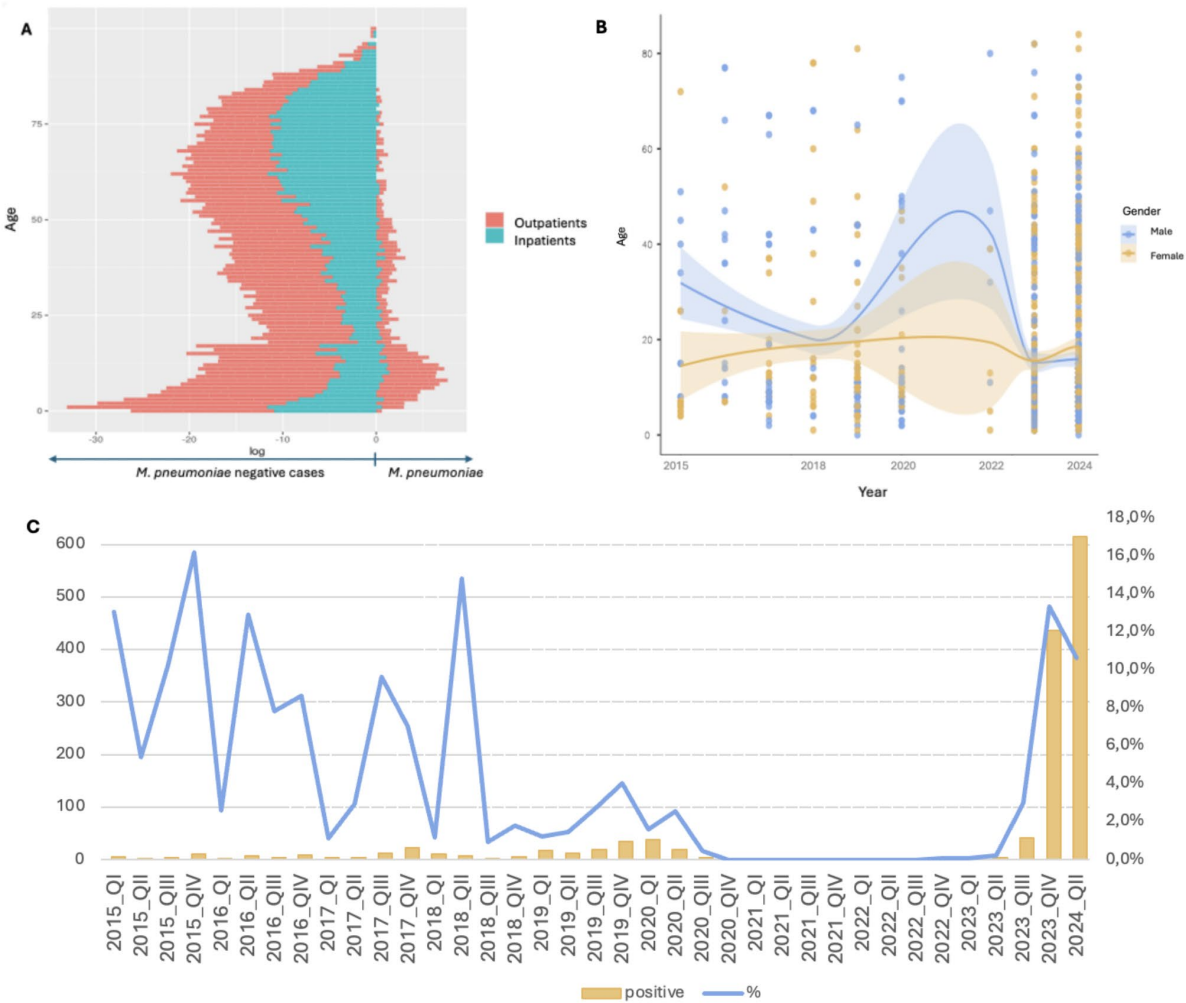
**A)** Age chart illustrating the different distribution of age and number of outpatients in *Mycoplasma pneumoniae* positive and negative cases. **B)** Distribution of age and sex in *Mycoplasma pneumoniae* positive cases from 2015–2024. **C)** Distribution of positive *Mycoplasma pneumoniae* tests and positivity rate in Germany from 2015–2024. Y-axis on the left-hand side showing overall number of positive *Mycoplasma pneumoniae* cases and on the right-hand side the percentage of positive *Mycoplasma pneumoniae* cases in comparison to all test for *Mycoplasma pneumoniae* performed. QI = January to March, QII = April to June, QIII = July to September, QIV = October to December

**Fig. 2:**
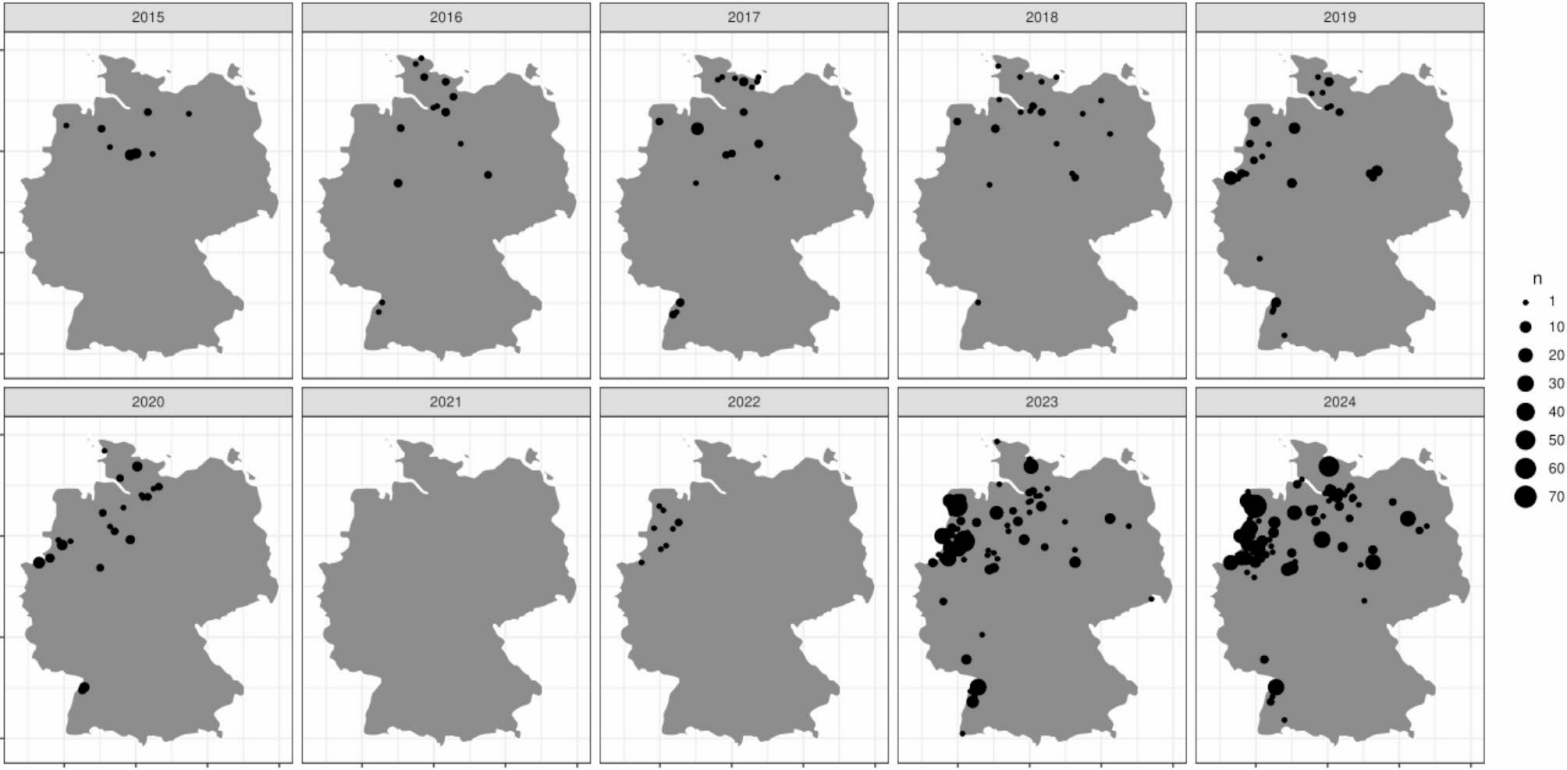
Geographical distribution of *Mycoplasma pneumoniae* cases from 2015–2024

We included 38,204 samples into the analysis. A total of 1448 cases (3.8%) were tested positive for *M. pneumoniae* (48.8% females). Baseline characteristics of *M. pneumoniae* patients are shown in Table 1. Pediatric patients (age ≤ 18 years) represented 75.7% of *M. pneumoniae* cases and 2.3% of patients were ≥ 60 years of age. *M. pneumoniae* positive cases were taken in 95.0% from outpatients compared to 78.4% in *M. pneumoniae* negative cases (MPN) (*p* < 0.001, aOR 0.56 95%-confidence interval (CI): 0.43–0.71) as illustrated in Fig. 1A. *M. pneumoniae* cases were significantly younger than MPN (median age 11 years (interquartile range (IQR) 8–17) vs. 42 years (IQR 9–65), *p* < 0.001, OR 0.98, 95%-CI 0.97–0.98). Hospitalized patients with *M. pneumoniae* were significantly older than outpatients (mean age 17 (IQR 11–40) vs. 11 years (IQR 7–16) in in- vs. outpatients respectively, *p* < 0.001, OR 1.03, 95% CI 1.01–1.04). We observed significant differences in age (*p* < 0.001, aOR 0.89 95%-CI 0.79–0.99) and sex (*p* = 0.029, aOR 0.98 (0.97,0.98)) in *M. pneumoniae* cases from 2015 to 2024 (Fig. 1B). Outpatient status (OR 0.56 95%-CI 0.43–0.71) and year of testing (OR dependent on year of testing, sTable 2) were further predictors of *M. pneumoniae* detection in multivariate analysis (*p* < 0.001). The regional distribution of *M. pneumoniae* cases over time shows a focus on the North and/or Western region of Germany (Fig. 2).

**Table 1:** Baseline characteristics of *Mycoplasma pneumoniae* positive patients. N = number of cases, IQR = interquartile range, na = not applicable

Baseline characteristics		Total	2015	2016	2017	2018	2019	2020	2021	2022	2023	2024
Total cases	*N*	1448	23	23	44	26	85	61	0	9	485	692
Age	Median (IQR)	11 (8–17)	7.5 (6–26)	24 (11–42)	9 (8–20)	14.5 (8–43.0)	10.0 (6–14)	10.0 (8–21)	na	32.0 (11–39)	10.5 (7–15)	11 (8–17)
Age groups												
0–18 years	N (%)	1090 (75.7)	15 (68.2)	9 (42.9)	30 (69.7)	16 (66.7)	67 (78.8)	44 (74.6)	na	4 (44.4)	387 (80.2)	518 (75.0)
19–59 years	N (%)	307 (21.3)	6 (27.3)	9 (42.9)	10 (23.3)	5 (20.8)	15 (17.6)	14 (23.7)	na	4 (44.4)	87 (18.0)	157 (22.8)
> 59 years	N (%)	42 (2.3)	1 (4.5)	3 (14.2)	3 (7.0)	3 (12.5)	3 (3.5)	1 (1.6)	na	1 (11.1)	8 (1.7)	15 (2.2)
Female sex	N (%)	690 (47.7)	15 (66.2)	5 (21.7)	19 (43.8)	16 (61.5)	45 (52.9)	24 (39.3)	na	6 (66.7)	244 (50.3)	317 (45.8)
Outpatients	N (%)	1375 (95.0)	22 (95.6)	18 (78.3)	40 (90.9)	22 (84.6)	74 (87.1)	55 (90.2)	na	8 (88.9)	469 (96.7)	667 (96.4)

Distribution of *M. pneumoniae* cases showed 225 (15.5%) of detected cased before and 1175 (81.1%) after COVID-19 pandemic (Table 2). In total, only 48 cases were detected during COVID-19 pandemic. While no difference in age and sex before and after COVID-19 pandemic were seen, we observed a significant increase in outpatients after COVID-19 pandemic (86.7 vs. 96.5%, *p* = < 0.001, aOR 0.25, 95% CI 0.15–0.4).

**Table 2:** *Mycoplasma pneumoniae* positive patients pre- and post-COVID-19 pandemic. *M. pneumoniae = Mycoplasma pneumoniae*. AOR = adjusted odd`s ratio, N = number of cases, IQR = interquartile range

Characteristics		*M. pneumoniae* pre-COVID-19 pandemic	*M. pneumoniae* post-COVID-19 pandemic	*p*-value (univariate analysis)	*p*-value (multivariate analysis)	aOR (95% CI)
**Age**	Median (IQR)	11 (7–24)	11 (8–16)	0.66	0.06	0.99 (0.98–1.0)
**Age groups**				0.30	-	-
0–18 years	N (%)	157 (69.8)	905 (77.0)			
19–59 years	N (%)	48 (21.3)	243 (20.7)			
> 59 years	N (%)	16 (7.1)	23 (2.0)			
**Female sex**	N (%)	111 (49.3)	561 (47.7)	0.66	0.73	1.05 (0.79–1.41)
**Outpatients**	N (%)	195 (86.7)	1134 (96.5)	< 0.001	< 0.001	0.25 (0.15–0.40)
**Total**	N (%)	225 (15.5)	1175 (81.1)	-	-	-

Highest positivity rates of *M. pneumoniae* were observed in November and December 2015 (16.2%), April to June 2016 (12.5%) and April to June 2018 (14.8%) and October to December 2023 (13.4%), while no cases of *M. pneumoniae* were detected in 2021 (Fig. 1C). An increase in overall cases was also detected in January to March 2024. Test ordering capacity significantly increased over time (*p* < 0.001).

Co-infections of *M. pneumoniae* with bacterial or viral pathogens were frequently detected (21.3%) from 2015 to 2024. Most common co-infections were influenza virus A/B, rhinovirus, metapneumovirus and respiratory syncytial virus (supplementary Table 1). We did not detect co-infections with common pathogens of CAP like *Streptococcus pneumoniae* and SARS-Cov-2 after introduction in PCR panel after 2022.

## Discussion

Our study highlights the epidemic spread of *M. pneumoniae* during the winter season 2015 and spring / summer 2016 and 2018 in Germany. Re-emergence of *M. pneumoniae* after COVID-19 pandemic was detected starting in October 2023 until the first quarter of 2024. *M. pneumoniae* cases showed a steep increase in comparison with the pre-pandemic number of cases in Germany. The positivity rate stayed the same even though testing capacity dramatically increased. Cases in the second quartile 2024 were decreasing. In contrast to other bacterial pathogens like *Streptococcus pyogene* [11], which showed increased clinical severity or *Streptococcus pneumoniae* which caused outbreaks after the COVID-19 pandemic, current spread of *M. pneumoniae* has exceeded the historical number of cases but this was mainly driven by outpatients suggesting mild clinical cases. Interestingly, most *M. pneumoniae* cases were young and not hospitalized, but hospitalized *M. pneumoniae* patients were significantly older. Similar characteristics were reported by a large scale outbreak in Marseille, France [12]. This contrasts current data from the Netherlands and Denmark [6, 7] who frequently observed hospitalization and 11% had severe *M. pneumoniae* CAP which needed treatment in the intensive-care unit. Severe *M. pneumoniae* CAP was reported by other European countries especially in adult patients since 2023 including Switzerland [13] and North-Western France [14].

Current empirical antibiotic treatment of CAP with beta-lactam with or without beta-lactamase-inhibitor is not active for *M. pneumoniae.* Treatment options include tetracycline, quinolones and macrolides. Addition of a macrolide to empirical treatment of CAP is currently limited by guidelines to severely ill hospitalized patients with CAP. Current treatment algorithms will withhold active antibiotic treatment in most cases as shown by our data as patients were mainly observed in the outpatient setting. Unfortunately, proportions of macrolide-resistant *M. pneumoniae* are constantly rising and have increased especially in the Western Pacific Area from 18.2% in 2000 to 76.5% in 2019 [15]. Current outbreak in China is reported to be dominated by a macrolide resistant clone [16]. Macrolide resistance is less pronounced in Europe with 5% resistant isolates. On the basis of this data we advise for the introduction of a reporting obligation in Germany to early detect epidemics and adjust of empirical antimicrobial treatment for in- and outpatients during epidemics of *M. pneumoniae* and early detect the development of antimicrobial resistance.

Our data is in line with previously reported epidemics of *M. pneumoniae* occurring every 3–7 years. The most plausible explanation is that in contrast to many other bacterial infections the adaptive immune system plays a central role for protections. Pre-existing high titers of Mycoplasma-specific IgG have been shown to be protective of *M. pneumoniae* infection in non-smokers [17, 18], but titers of IgG antibodies decline over time. In contrast, during the COVID-19 pandemic low numbers of *M. pneumoniae* cases have been reported [8, 19]. Non-pharmaceutical interventions (NPI) have been shown to be protective of SARS-Cov-2 infection and infection with other respiratory viruses [20] and have also been also protective for *M. pneumoniae* [19]. Since infections with *M. pneumoniae* are distributed by droplets like other respiratory viruses and SARS-Cov-2 NPI must have had an effect on *M. pneumoniae* distribution in the population during COVID-19 pandemic. This might have led to declining antibody titers in the population, increased susceptibility and current *M. pneumoniae* epidemic in Germany [8].

Furthermore, co-infection was observed in every fifth patient with influenza virus A/B and rhinovirus being most prevalent. Rhinovirus was the most prevalent co-infection in a *M. pneumoniae* outbreak in France in 2024, especially in children < 5 years [12]. Data from China showed an adenovirus epidemic coinciding with the *M. pneumoniae* outbreak, which ended in the first quarter of 2024 [9]. Co-infections with influenza virus was also common after COVID-19 pandemic [21]. However, the data did not allow us to analyze the influence of possible co-infections systematically. This cohort is characterized by a high number of pediatric outpatients which confirms clinical data on *M. pneumoniae* CAP to be less severe. Adult patients were the minority of *M. pneumoniae* cases in most seasons in our cohort. This contrasts recent data from Denmark where 7% of children, 19% of adult patients aged 19–74 years and 48% >75 years were hospitalized over several seasons [6]. *M. pneumoniae* was reported to be among the five most common pathogens of severe CAP [4]. Current German guidelines on CAP advise against routine testing for *M. pneumoniae* in adults and children. Therefore, the following hypothesis can be raised which should be targeted by future studies: (I) Pediatricians are more aware of *M. pneumoniae* CAP. (II) Adult CAP patients are highly underdiagnosed with *M. pneumoniae* in Germany.

Our study has some limitations: No information on clinical disease and course of infections was available. Long-term colonization and asymptomatic carriers with *M. pneumoniae* have been reported [22], but should then also have been detected during the Covid-19 pandemic. Since PCR diagnostics are expensive we assume that treating physicians only initiate diagnostics in symptomatic patients. Also, higher bacterial load has been reported in symptomatic patients. The majority of samples were taken from outpatient children, which suggests the benign nature of the clinical pictures encountered. We cannot measure the rate of secondary hospitalizations of patients which might have led to an overestimation of outpatients in our cohort. Furthermore, we cannot estimate incidence of *M. pneumoniae* in all areas of Germany since most patients were localized in the North and/or West of Germany (Fig. 2). An epidemic situation of *M. pneumoniae* infections has been reported by adjacent countries in 2023/2024 which suggests generalizability of our results [6, 7]. Detection of pathogens tested outside of the panels described was not included into the analysis. Therefore, the rate of coinfection could be higher, especially with SARS-Cov-2.

In conclusion, empirical treatment of CAP patients often does not include coverage of *M. pneumoniae*. Based on a more thorough implementation of available surveillance data into clinical routine, respective therapies could be adapted more quickly during epidemic outbreaks of *M. pneumoniae* infections. As hospitalization is increased in adult patients and severe courses of disease have been frequently reported, physicians should be aware and test for *M. pneumoniae* in CAP.

## Electronic supplementary material

Below is the link to the electronic supplementary material.

Supplementary Material 1

## Data Availability

The datasets used and/or analysed during the current study are available from the corresponding author upon request.

## Abbreviations

95%-CI: 95% confidence interval
CAP: Community acquired pneumonia
IQR: Interquartile range
MPN: *M. pneumoniae* negative cases
OR: Odd’s ratio
PCR: Polymerase chain reaction

## References

[1] Kutty PK, Jain S, Taylor TH, Bramley AM, Diaz MH, Ampofo K, Arnold SR, Williams DJ, Edwards KM, McCullers JA, Pavia AT, Winchell JM, Schrag SJ, Hicks LA. Mycoplasma pneumoniae among children hospitalized with community-acquired Pneumonia. Clin Infect Dis. 2019;68:5–12. 10.1093/cid/ciy419. 29788037 10.1093/cid/ciy419 PMC6552676

[2] Principi N, Esposito S, Blasi F, Allegra L, group M study. Role of Mycoplasma pneumoniae and Chlamydia pneumoniae in children with Community-Acquired Lower Respiratory Tract infections. Clin Infect Dis. 2001;32:1281–9. 10.1086/319981. 11303262 10.1086/319981

[3] Beeton ML, Zhang X-S, Uldum SA, Bébéar C, Dumke R, Gullsby K, Ieven M, Loens K, Nir-Paz R, Pereyre S, Spiller OB, Chalker VJ. Subgroup ESG for M and CI (ESGMAC) M pneumoniae, author ESG for M and CI (ESGMAC) M pneumoniae subgroup members not listed as an individual (2020) Mycoplasma pneumoniae infections, 11 countries in Europe and Israel, 2011 to 2016. Eurosurveillance 25:1900112. 10.2807/1560-7917.es.2020.25.2.1900112 10.2807/1560-7917.ES.2020.25.2.1900112 PMC6976882 31964459

[4] Qu J, Zhang J, Chen Y, Huang Y, Xie Y, Zhou M, Li Y, Shi D, Xu J, Wang Q, He B, Shen N, Cao B, She D, Shi Y, Su X, Zhou H, Fan H, Ye F, Zhang Q, Tian X, Lai G. Aetiology of severe community acquired pneumonia in adults identified by combined detection methods: a multi-centre prospective study in China. Emerg Microbes Infect. 2022;11:556–66. 10.1080/22221751.2022.2035194. 35081880 10.1080/22221751.2022.2035194 PMC8843176

[5] Narita M. Classification of Extrapulmonary manifestations due to Mycoplasma pneumoniae infection on the basis of possible pathogenesis. Front Microbiol. 2016;7:23. 10.3389/fmicb.2016.00023. 26858701 10.3389/fmicb.2016.00023 PMC4729911

[6] Nordholm AC, Søborg B, Jokelainen P, Møller KL, Sørensen LF, Krause TG, Uldum SA, Emborg H-D. Mycoplasma pneumoniae epidemic in Denmark, October to December, 2023. Eurosurveillance. 2024;29:2300707. 10.2807/1560-7917.es.2024.29.2.2300707. 38214084 10.2807/1560-7917.ES.2024.29.2.2300707 PMC10785206

[7] Bolluyt DC, Euser SM, Souverein D, van Rossum AM, Kalpoe J, van Westreenen M, Goeijenbier M, Snijders D, Eggink D, Jongenotter F, van Lelyveld SF, van Houten MA. (2024) Increased incidence of Mycoplasma pneumoniae infections and hospital admissions in the Netherlands, November to December 2023. Eurosurveillance 29:. 10.2807/1560-7917.es.2024.29.4.2300724 10.2807/1560-7917.ES.2024.29.4.2300724 PMC10986650 38275014

[8] Sauteur PMM, Beeton ML, group ES of CM and ID (ESCMID) SG for M and CI (ESGMAC) and the ESGMAC Mycoplasma pneumoniae Surveillance (MAPS) study, Pereyre S, Bébéar C, Gardette M, Hénin N, Wagner N, Fischer A, Vitale A, Lemaire B, Greub G, Brouillet R, Zimmermann P, Agyeman PK, Aebi C, Buettcher M, Hostettler M, Kottanattu L, Gaia V, Imkamp F, Egli A, Berger C, Sidorov S, Osuna E, Tilen R, Niederer-Loher A, Dollenmaier G, Barbey F, Heininger U, Goldenberger D, Ivan B, Keller PM, Papan C, Becker SL, Forster J, MacKenzie CR, Henrich B, Vermeulen M, Bossuyt N, Matheeussen V, van Westreenen M, Vogel M, van Rossum AM, Afshar B, Cottrell S, Moore C, Uldum SA, Emborg H-D, Gullsby K, Laine M, Peltola V, Heinonen S, Døllner H, Width FG, Christensen A, Buonsenso D, Rodrigues FMP, Rodrigues J, Tsantila K, Matsas M, Kalogera E, Petridou E, Kopsidas I, Keše D, Nir-Paz R, Elinav H, Michael-Gayego A, Oishi T, Saraya T, Kenri T, Hsieh Y-C, Wu T-H, Maiwald M, Loo LH, Sagar T, Chaudhry R, Kociolek LK, Kies KD, Mainella J, Kapinos J, Patel R, Rodríguez N, Lorenz D, Blakiston MR. Mycoplasma pneumoniae: delayed re-emergence after COVID-19 pandemic restrictions. Lancet Microbe. 2024;5:e100–1. 10.1016/s2666-5247(23)00344-0. 38008103 10.1016/S2666-5247(23)00344-0

[9] Wang W, Luo X, Ren Z, Fu X, Chen Y, Wang W, Bao Y, Zheng Y, Cao K, Chen J. Impact of COVID-19 pandemic measures on hospitalizations and epidemiological patterns of twelve respiratory pathogens in children with acute respiratory infections in southern China. BMC Infect Dis. 2025;25(1):103. 10.1186/s12879-025-10463-y. 39844061 10.1186/s12879-025-10463-y PMC11756097

[10] Dumke R. The high-incidence period of Mycoplasma pneumoniae infections 2023/2024 in southeast Germany was associated with a low level of macrolide resistance. Infection. 2024;52(6):2525–7. 10.1007/s15010-024-02336-4. 38949755 10.1007/s15010-024-02336-4 PMC11621181

[11] Alcolea-Medina A, Snell LB, Alder C, Charalampous T, Williams TGS, Group SML, Athitha V, Begum J, Bonaiti M, Brennan J, Bryan L, Cerda A, Cliff PR, Hoang LH, Merrill TV, Naumova D, Parvez R, Valle K, White S, Wray D, Tan MKI, Al-Yaakoubi N, Humayun G, Newsholme W, Goldenberg S, Nebbia G, Neil SJD, Batra R, Edgeworth JD. The ongoing Streptococcus pyogenes (Group A Streptococcus) outbreak in London, United Kingdom, in December 2022: a molecular epidemiology study. Clin Microbiol Infect. 2023;29:887–90. 10.1016/j.cmi.2023.03.001. 36925107 10.1016/j.cmi.2023.03.001 PMC10769882

[12] Edouard S, Boughammoura H, Colson P, La Scola B, Fournier PE, Fenollar F. Large-scale outbreak of Mycoplasma pneumoniae infection, Marseille, France, 2023–2024. Emerg Infect Dis. 2024;30(7):1481–4. 10.3201/eid3007.240315. 38816344 10.3201/eid3007.240315 PMC11210650

[13] Garzoni C, Bernasconi E, Zehnder C, Malossa SF, Merlani G, Bongiovanni M. Unexpected increase of severe Mycoplasma pneumoniae pneumonia in adults in Southern Switzerland. Clin Microbiol Infection: Official Publication Eur Soc Clin Microbiol Infect Dis. 2024;30(7):953–4. 10.1016/j.cmi.2024.03.008. 10.1016/j.cmi.2024.03.008 38461940

[14] Zayet S, Poloni S, Plantin J, Hamani A, Meckert Y, Lavoignet CE, Gendrin V, Klopfenstein T. Outbreak of *Mycoplasma pneumoniae* pneumonia in hospitalized patients: who is concerned? Nord Franche-Comté Hospital, France, 2023–2024. Epidemiol Infect. 2024;152:e46. 10.1017/S0950268824000281. 38356388 10.1017/S0950268824000281 PMC10983049

[15] Kim K, Jung S, Kim M, Park S, Yang H-J, Lee E. Global trends in the proportion of Macrolide-Resistant Mycoplasma pneumoniae infections. JAMA Netw Open. 2022;5:e2220949. 10.1001/jamanetworkopen.2022.20949. 35816304 10.1001/jamanetworkopen.2022.20949 PMC9274321

[16] Li H, Li S, Yang H, Chen Z, Zhou Z. Resurgence of Mycoplasma pneumonia by macrolide-resistant epidemic clones in China. Lancet Microbe. 2024;5(6):e515. 10.1016/S2666-5247(23)00405-6. 38244553 10.1016/S2666-5247(23)00405-6

[17] Klement E, Talkington DF, Wasserzug O, Kayouf R, Davidovitch N, Dumke R, Bar-Zeev Y, Ron M, Boxman J, Thacker WL, Wolf D, Lazarovich T, Shemer-Avni Y, Glikman D, Jacobs E, Grotto I, Block C, Nir-Paz R. Identification of risk factors for infection in an outbreak of Mycoplasma pneumoniae respiratory Tract Disease. Clin Infect Dis. 2006;43:1239–45. 10.1086/508458. 17051486 10.1086/508458

[18] de Groot RCA, Estevão SC, Sauteur PMM, Perkasa A, Hoogenboezem T, Spuesens EBM, Verhagen LM, van Rossum AMC, Unger WWJ. Mycoplasma pneumoniae carriage evades induction of protective mucosal antibodies. Eur Respir J. 2022;59:2100129. 10.1183/13993003.00129-2021. 34561284 10.1183/13993003.00129-2021 PMC8989055

[19] Sauteur PMM, Beeton ML, Uldum SA, Bossuyt N, Vermeulen M, Loens K, Pereyre S, Bébéar C, Keše D, Day J, Afshar B, Chalker VJ, Greub G, Nir-Paz R, Dumke R, Wagner N, Andreutti C, Agyeman PKA, Aebi C, Buettcher M, Kottanattu L, Gaia V, Imkamp F, Zbinden R, Berger C, Niederer-Loher A, Barbey F, Egli A, Schmid H, Heininger U, Papan C, Vasconcelos MK, Henrich B, Mackenzie C, Schneider G, van Westreenen M, Verkaik NJ, van Rossum AMC, Emborg H-D, Peltola V, Renko M, Tapiainen T, Heinonen S, Døllner H, Rodrigues F, Matsas M, Kalogera E, Petridou E, Kopsidas I, Zaoutis TE, Michael-Gayego A, Ouchi K, Namkoong H, Hsieh Y-C, Maiwald M, Loo LH, Chaudhry R, Kociolek LK, Rodríguez N, Lorenz D, Almeida MD. Mycoplasma pneumoniae detections before and during the COVID-19 pandemic: results of a global survey, 2017 to 2021. Eurosurveillance. 2022;27:2100746. 10.2807/1560-7917.es.2022.27.19.2100746. 35551702 10.2807/1560-7917.ES.2022.27.19.2100746 PMC9101966

[20] Käding N, Waldeck F, Meier B, Boutin S, Borsche M, Balck A, Föh B, Kramer J, Klein C, Katalinic A, Rupp J. Influence of non-pharmaceutical interventions during the COVID-19 pandemic on respiratory viral infections– a prospective population-based cohort study. Front Public Heal. 2024;12:1415778. 10.3389/fpubh.2024.1415778. 10.3389/fpubh.2024.1415778 PMC11228307 38979040

[21] Zhang Y, Su C, Zhang Y, Ding S, Yan X, Zhang J, Tao Z. Epidemiological and clinical characteristics of hospitalized pediatric patients with Mycoplasma pneumoniae pneumonia before and after the COVID-19 pandemic in China: a retrospective multicenter study. BMC Infect Dis. 2025;25(1):18. 10.1186/s12879-024-10370-8. 39754040 10.1186/s12879-024-10370-8 PMC11699690

[22] Spuesens EB, Fraaij PL, Visser EG, Hoogenboezem T, Hop WC, van Adrichem LN, Weber F, Moll HA, Broekman B, Berger MY, van Rijsoort-Vos T, van Belkum A, Schutten M, Pas SD, Osterhaus AD, Hartwig NG, Vink C, van Rossum AM. Carriage of Mycoplasma pneumoniae in the upper respiratory tract of symptomatic and asymptomatic children: an observational study. PLoS Med. 2013;10(5):e1001444. 10.1371/journal.pmed.1001444. 23690754 10.1371/journal.pmed.1001444 PMC3653782

