# Supplementary material for: Re-emergence of Mycoplasma pneumoniae before and after COVID-19 pandemic in Germany
Source: BMC Infect Dis. 2025 Mar 6;25:318. doi: 10.1186/s12879-025-10657-4 (PMC11884198; doi:10.1186/s12879-025-10657-4)
Supplement: Supplementary file 1 — Supplementary Material 1 [file 12879_2025_10657_MOESM1_ESM.docx]

**Supplementary material**

sTable 1: Multiplex and targeted PCRs used for testing of *Mycoplasma pneumoniae* and other respiratory viral and bacterial pathogens. x = testing performed by this PCR, *testing since 2022.

| **Pathogens** | **Panel 1** | **Panel 2** | **targeted**  ***M. pneumoniae* PCR** | **positive Tests** |
| --- | --- | --- | --- | --- |
| Viral pathogens:   - Adenovirus | x | x |  | 26 |
| - Human bocavirus |  | x |  | 0 |
| - Coronavirus (229E, HKU1, NL63, OC43) |  | x |  | 14 |
| - Influenza virus A/B | x | x |  | 92 |
| - Human Metapneumovirus | x | x |  | 39 |
| - Parainfluenza virus 1-4 | x | x |  | 8 |
| - Rhino-/Enterovirus |  | x |  | 19 |
| - Human rhinovirus A/B/C | x |  |  | 44 |
| - Respiratory syncytial virus | x | x |  | 29 |
| - SARS-Cov-2* |  | x |  | 0 |
| Bacterial pathogens:   - *Bordetella pertussis / parapertussis* | x |  |  | 17 |
| - *Chlamydophila pneumoniae* | x | x |  | 18 |
| - *Haemophilus influenzae* | x |  |  | 3 |
| - *Mycoplasma pneumoniae* | x | x | x | 1448 |
| - *Streptococcus pneumoniae* | x |  |  | 0 |
| - *Legionella pneumophila* | x | x |  | 0 |
|  |  |  |  |  |
| **Total tests** | 7874 | 24898 | 5432 | 1757 |

sTable2: Logistic regression predicting *Mycoplasma pneumoniae.* OR= odd’s ratio, 95-CI = 95% confidence interval.

| **Characteristic** | **Crude**  **OR (95%-CI)** | **Adjusted**  **OR (95%-CI)** | **P-value**  **(Wald’s test)** | **p-value**  **(LR-test)** |
| --- | --- | --- | --- | --- |
| Gender (reference female) | 0.95 (0.85, 1.05) | 0.89 (0.79, 0.99) | 0.029 | 0.029 |
| Age | 0.98 (0.97, 0.98) | 0.98 (0.97, 0.98) | < 0.001 | < 0.001 |
| Inpatient status | 0.2 (0.15, 0.25) | 0.56 (0.43, 0.71) | < 0.001 | < 0.001 |
| **Year of testing** (reference 2015) |  |  |  | < 0.001 |
| 2016 | 0.61 (0.33, 1.14) | 0.66 (0.34, 1.25) | 0.2 |  |
| 2017 | 0.39 (0.23, 0.67) | 0.24 (0.14, 0.42) | < 0.001 |  |
| 2018 | 0.13 (0.07, 0.24) | 0.07 (0.04, 0.13) | < 0.001 |  |
| 2019 | 0.18 (0.11, 0.29) | 0.12 (0.07, 0.19) | < 0.001 |  |
| 2020 | 0.11 (0.07, 0.19) | 0.08 (0.05, 0.13) | < 0.001 |  |
| 2021 | 0 (0, 2.8e+87) | 0 (0, 8.9e+141) | 0.92 |  |
| 2022 | 0.01 (0.01, 0.03) | 0.01 (0, 0.02) | < 0.001 |  |
| 2023 | 0.43 (0.28, 0.68) | 0.3 (0.19, 0.48) | < 0.001 |  |
| 2024 | 0.86 (0.55, 1.34) | 0.56 (0.35, 0.9) | 0.016 |  |
